# Bacterial aerotaxis: receptor diversity, signal transduction and ecological functions

**DOI:** 10.7717/peerj.21573

**Published:** 2026-07-22

**Authors:** Zhiwei Huang, Wanpeng Zhang, Chengning Qin, Hongliang Zhao, Feiyu Yan, Guoliang Zhang

**Affiliations:** 1 College of Life Science and Food Engineering/College of Agriculture, Huaiʻan University, Huaiʻan, Jiangsu Province, China; 2 Jiangsu Provincial Agricultural Green and Low Carbon Production Technology Engineering Research Center, Huaiʻan University, Huaiʻan, Jiangsu Province, China

**Keywords:** Aerotaxis, Receptors, Signal transduction, Adaptation mechanism, Host-microbe interactions

## Abstract

Aerotaxis, the oxygen-directed motile behavior intrinsically linked to bacterial energy metabolism, serves as a fundamental survival and adaptive strategy for motile prokaryotes, including bacteria and archaea. This behavior enables these microorganisms to navigate along oxygen gradients and colonize metabolically optimal ecological niches. This review systematically synthesizes the latest research advancements in bacterial aerotaxis, focusing on the diverse signal-sensing mechanisms of aerotaxis receptors, which are primarily categorized into energy-dependent and direct oxygen-sensing types. Furthermore, we elucidate the distinct molecular mechanisms that underlie their oxygen signal perception and transduction. We also discuss the two major adaptive regulatory mechanisms of aerotaxis and its critical ecological functions in mediating microbe-host interactions, facilitating host colonization, and modulating biofilm formation. This work clarifies the evolutionary and ecological significance of aerotaxis in prokaryotic environmental adaptation and provides a comprehensive theoretical framework for future relevant research.

## Introduction

The natural environment is complex and variable, presenting ongoing challenges to the survival and proliferation of microorganisms. In response to these challenges, microbes have evolved a diverse array of mechanisms to adapt to fluctuations in various environmental factors. Signal transduction serves as the primary mechanism by which bacteria detect and respond to environmental stimuli. Among the various signal transduction mechanisms, taxis has been extensively studied in bacteria (Krell et al., 2011; Liu et al., 2025). Taxis refers to the ability of motile bacteria to navigate through gradients of diverse physicochemical factors (Alexandre, Greer-Phillips & Zhulin, 2004). It can be further categorized into chemotaxis, phototaxis, aerotaxis, thermotaxis, pH taxis, osmotaxis, and magnetotaxis, based on microbial responses to specific environmental cues, such as chemicals, light, oxygen, temperature, pH, osmolarity, and magnetic fields (Armitage, 1997; Taylor, Zhulin & Johnson, 1999; Paulick et al., 2017; Rosko et al., 2017; Perlova et al., 2019; Tohidifar et al., 2020; Keegstra, Carrara & Stocker, 2022; Herz et al., 2025). Aerotaxis is one of the most extensively studied taxis next to chemotaxis, and its molecular mechanism is closely linked to bacterial energy metabolism (Taylor, Zhulin & Johnson, 1999; Alexandre, 2010; Schweinitzer & Josenhans, 2010).

Oxygen is a critical environmental factor that influences bacterial metabolism, behavior, and spatial distribution in natural habitats. Aerotaxis enables bacteria to detect fluctuations in oxygen concentration, allowing them to seek ecological niches that are conducive to growth and energy metabolism (Taylor, Zhulin & Johnson, 1999; Schweinitzer & Josenhans, 2010). The aerotactic behavior of bacteria was first documented in 1881 by Engelmann, who observed the aggregation of bacteria around air bubbles at the edges of coverslips and around oxygen-producing plant cells (Engelmann, 1881). Beijerinck (1893) investigated the distribution of bacteria beneath a coverslip and identified three distinct aerotactic response types: aerobic bacteria migrated toward the air–liquid interface, anaerobic bacteria moved maximally away from the interface, and microaerophiles accumulated at a short distance from the oxygen source. Building on these early observations, Baracchini & Sherris (1959) tested 24 motile aerobic and facultatively anaerobic bacterial species, demonstrating that all exhibited positive aerotaxis, while three anaerobic species displayed negative aerotaxis. Following the 1960s, aerotaxis has received increasing attention from scholars. The aerotactic behaviors of *Escherichia coli*, *Spirillum volutans*, and *Azospirillum brasilense* have been characterized through the observation of the formation and changes of aerotactic bands in capillary tubes (Adler, 1966; Caraway & Kreig, 1974; Barak et al., 1982). Studies revealed that the aerotactic bands formation by *S. volutans* cells requires oxidizable substrates, inorganic ions, and chelating agents (Caraway & Kreig, 1974). While the formation of aerotactic bands in *A. brasilense* is regulated by extracellular pH, cell density, culture age, incubation duration, and respiratory inhibitors (Barak et al., 1982). The position of the aerotactic bands in capillaries correlates with cellular respiration rate and oxygen concentration (Caraway & Kreig, 1974; Barak et al., 1982). These studies mark the beginning of a comprehensive investigation into aerotaxis. During this period, aerotaxis was confirmed to be a distinct behavioral process from chemotaxis: unlike chemotaxis, which is independent of electron transport, aerotaxis signal transduction is dependent on this process (Laszlo & Taylor, 1981; Laszlo et al., 1984). Subsequent research identified a close association between proton motive force (PMF) and aerotaxis (Zhulin et al., 1996). In *E. coli*, Aer and the serine chemoreceptor Tsr were shown to independently sense intracellular energy levels and transduce signals related to oxygen, redox states, and energy to modulate bacterial behavior (Rebbapragada et al., 1997). Aerotaxis is increasingly recognized as a form of energy taxis (Taylor, Zhulin & Johnson, 1999). In fact, energy taxis is a broad concept that encompasses various directed movements, including aerotaxis, phototaxis, redox taxis, and chemotaxis toward oxidizable substrates (Taylor, Zhulin & Johnson, 1999; Schweinitzer & Josenhans, 2010). However, the discovery of HemAT, a novel class of aerotaxis receptors that directly bind oxygen, provided new insights into aerotaxis mechanisms (Hou et al., 2000; Hou et al., 2001a). To date, the signal transduction mechanisms of aerotaxis have been characterized in greater detail. Researchers have identified and investigated aerotaxis across various bacterial species (Taylor, Zhulin & Johnson, 1999; Nichols & Harwood, 2000; Alexandre, 2010; Schweinitzer & Josenhans, 2010; Armitano, Méjean & Jourlin-Castelli, 2013; Jiang et al., 2016; Greer-Phillips et al., 2018; Arrebola & Cazorla, 2020; Orillard et al., 2021; Stuffle et al., 2022; Tumewu et al., 2022; Ichinose et al., 2023; Huang et al., 2024; Herz et al., 2025). Research has also revealed that aerotaxis functions extend far beyond simple oxygen sensing behavior: it is closely linked to bacterial pathogenicity (García-Fontana et al., 2019; Ichinose et al., 2023; Huang et al., 2024), efficient host adhesion and colonization (Greer-Phillips, Stephens & Alexandre, 2004; Jiang et al., 2016; O’Neal, Akhter & Alexandre, 2019; Arrebola & Cazorla, 2020; Ganusova et al., 2023), and bacterial magnetotaxis (Herz et al., 2025).

Despite the increasing number of studies on aerotaxis, the signaling mechanisms involving various types of receptors in aerotaxis and related physiological processes remain inadequately elucidated. Furthermore, the functional differentiation of multiple aerotaxis receptors within a single strain is not well understood. This review systematically elucidates the oxygen signal transduction mechanisms of different types of aerotaxis receptors, analyzes their structural diversity, and defines their roles within the signaling systems. Additionally, the review discusses the adaptive mechanisms of aerotaxis and emphasizes its importance for microbial survival by exploring the ecological roles of aerotaxis. This aims to provide a reference for future research on the function of aerotaxis receptors by systematically integrating current advancements in the field.

## Search Methodology

The data and research findings synthesized in this review were obtained through a rigorous literature search across multiple authoritative academic databases, including PubMed, Google Scholar, and Web of Science. A structured Boolean search strategy was adopted with core keywords closely related to bacterial aerotaxis, including aerotaxis, bacterial aerotaxis, aerotaxis receptor, energy taxis, oxygen sensor, oxygen sensing, PAS domain, globin domain, biofilm formation, and host-microbe interaction. The time frame for the literature retrieval spanned from January 1881 to March 2026. Literature screening followed strict inclusion criteria: only English original research articles, peer-reviewed reviews, and short communications from reputable journals were included. Screening was completed in two stages: initial manual screening of titles and abstracts for relevance, followed by full-text evaluation for scientific rigor, novelty, and suitability for this review. EndNote was used for literature management and synthesis. This multi-stage process ensured the inclusion of high-quality, relevant research, guaranteeing the reliability and accuracy of the review conclusions.

### Classical signal transduction system in bacterial taxis

In prokaryotes, the detection of environmental cues is primarily facilitated by two-component systems (TCS), which generally comprise a sensor histidine kinase and a phosphorylatable response regulator (Huang et al., 2018; Gumerov, Andrianova & Zhulin, 2021; Liu et al., 2025). The chemotaxis system is a special case of TCS that has been extensively studied in *E. coli* (Parkinson, Hazelbauer & Falke, 2015; Colin & Sourjik, 2017; Gumerov, Andrianova & Zhulin, 2021). Within the chemotaxis system, the sensor histidine kinase is differentiated into three core components: the chemoreceptor, also known as the methyl-accepting chemotaxis protein (MCP), the coupling protein, and the histidine kinase itself (Liu et al., 2025). The *E. coli* chemotaxis system possesses four transmembrane chemoreceptors, specifically Tsr, Tar, Tap, and Trg, as well as one membrane-bound cytoplasmic receptor Aer (Alexandre, Greer-Phillips & Zhulin, 2004; Parkinson, Hazelbauer & Falke, 2015). Aer serves as the principal receptor for sensing intracellular energy levels and mediating signal transduction related to aerotaxis and other energy-dependent behaviors, whereas Tsr is also capable of detecting signals associated with oxygen (Bibikov et al., 1997; Rebbapragada et al., 1997). In the chemotaxis system, two coupling protein CheW monomers couple one histidine autokinase CheA dimer to two chemoreceptor trimers of dimers, thereby forming a core signaling unit (CSU) at the cell pole (Tran et al., 2023). Numerous CSUs form a large chemosensory array that plays a pivotal role in detecting environmental cues and transmitting signals across the cell membrane into the cytoplasmic space (Cassidy et al., 2023; Tran et al., 2023; Liu et al., 2025). The phosphorylatable response regulator within chemotaxis system, CheY, is a cytoplasmic protein capable of being phosphorylated by CheA, thus facilitating downstream signal transduction. Given the roles of CheW, CheA, and CheY in oxygen-directed chemotaxis and the assembly of Aer into higher-order signaling complexes *via* interactions with CheA and CheW (Rowsell et al., 1995; Rebbapragada et al., 1997; Samanta et al., 2016), it is inferred that the CSU constitutes a conserved structural foundation for both chemotaxis and aerotaxis. Additionally, phosphorylated CheY serves as a universal output mechanism for signal transduction.

In the CSU, the detection and interaction with signal molecules are predominantly mediated by the ligand-binding domains (LBDs) of chemoreceptors (Bi & Sourjik, 2018). In the absence of signal molecule engagement with the LBD, the kinase CheA undergoes autophosphorylation, subsequently transferring phosphate groups to the response regulator CheY. The phosphorylated form of CheY interacts with the flagellar motor protein FliM, resulting in a shift of the flagellar rotation from counter-clockwise (CCW) to clockwise (CW), thereby leading to tumbling movement of the cell (Colin & Sourjik, 2017). Concurrently, the phosphatase CheZ facilitates the dephosphorylation of CheY, which terminates the signal that alters flagellar rotation, ultimately promoting forward swimming of the cell. When signal molecules bind to the LBD, a conformational change is induced in the chemoreceptor cytoplasmic domain, which inhibits CheA autophosphorylation activity. This leads to a reduction in intracellular phosphorylated CheY levels and sustained unidirectional bacterial movement (Parkinson, Hazelbauer & Falke, 2015; Bi & Sourjik, 2018). The chemotaxis incorporates an adaptation mechanism that enables cells to return to their prestimulus state following a transient response to a persistent signal. This mechanism involves the methylation and demethylation of specific glutamate residues within the cytoplasmic domain of the chemoreceptors, processes catalyzed by the methyltransferase CheR and the methylesterase CheB (Tran et al., 2023). Inhibition of the autophosphorylation activity of CheA prevents CheB from receiving phosphate groups from CheA, thereby hindering the demethylation of methylated chemoreceptors, while CheR continues to methylate the chemoreceptors (Huang et al., 2019a). This modulation alters the affinity of signaling molecules for the core signal complex, effectively restoring it to its prestimulus state (Parkinson, Hazelbauer & Falke, 2015; Tran et al., 2023). Such a dynamic regulatory mechanism ensures that bacteria can accurately detect gradient changes across a broad spectrum of chemical concentrations, thereby maintaining an efficient motility strategy.

### Diverse aerotaxis receptors and their signal transduction mechanisms

**Figure 1:**
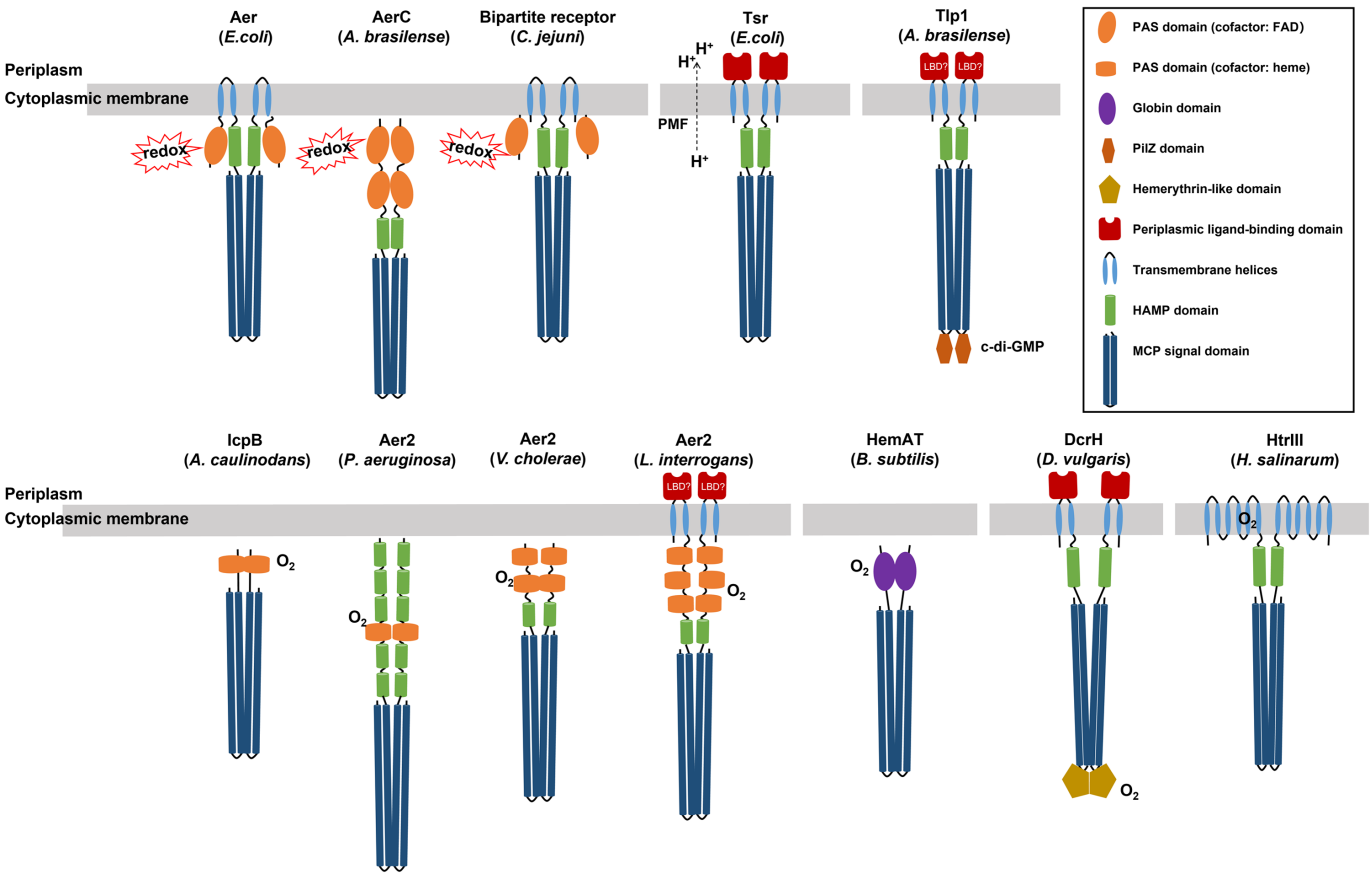
Structural diversity of bacterial and archaeal aerotaxis receptors and their oxygen-sensing mechanisms. The schematic representation provides an overview of representative aerotaxis receptors from a variety of prokaryotic organisms, highlighting their domain architectures and oxygen-sensing mechanisms. The receptors are categorized based on their primary sensing mechanisms: energy-dependent aerotaxis (top row) and direct oxygen sensing (bottom row). All receptors illustrated in the figure are presented in their dimeric forms. The names and origins of each receptor are annotated directly above their respective structures. The primary structural domains, along with the types and names of transmembrane helices that comprise the receptors, are elaborated in the legend located in the upper right corner of the image. Furthermore, the types of stimuli or signaling molecules recognized by the receptors are indicated adjacent to the corresponding structural domains responsible for signal perception or molecular binding. This figure was constructed using the drawing tools in Microsoft PowerPoint 2021, exported as a PNG file, and further cropped and resized to the appropriate dimensions using the Paint utility included with Windows 11.

The well-characterized classical *E. coli* chemotaxis system serves as a conserved molecular framework for bacterial taxis responses. However, in most bacteria, chemotaxis systems exhibit remarkable diversity, which is evident not only in the presence of homologous core components (*e.g.*, CheW, CheY) and novel non-core proteins (*e.g.*, CheD, CheT), but also in the variety and quantity of chemoreceptors (Bi & Sourjik, 2018; Huang et al., 2019a; Gao et al., 2021; Agbekudzi et al., 2024; Huang et al., 2025; Liu et al., 2025). This diversity is particularly pronounced in aerotaxis receptors: current studies indicate that bacteria have evolved a diverse repertoire of aerotaxis receptors with specialized structural and functional properties, which mediate oxygen signal perception and transduction through mechanisms that either conform to or diverge from the classical system (Alexandre, 2010; Schweinitzer & Josenhans, 2010; Herz et al., 2025). Based on oxygen signal perception modes, aerotaxis receptors are generally categorized into two types. The first type, also known as energy taxis receptors, does not sense oxygen directly but monitors the cellular redox state of the electron transport chain or PMF to initiate an oxygen-sensing pathway (Zhulin et al., 1996; Rebbapragada et al., 1997; Edwards, Johnson & Taylor, 2006). These receptors rely on intracellular energy status changes to guide the direction of bacterial movement (Alexandre, 2010; Schweinitzer & Josenhans, 2010; Samanta et al., 2016). The second type mediates direct oxygen sensing through direct interaction between intracellular domains and molecular oxygen, thus exhibiting a distinct response mechanism compared to the energy taxis receptors (Hou et al., 2000; Jiang et al., 2016; Huang et al., 2024). The two types of aerotaxis receptors are described in detail in the following sections and Table S1. The receptors demonstrate typical structural characteristics and oxygen response mechanisms, as depicted in Fig. 1.

### Energy-dependent aerotaxis receptors

#### PAS domain-coupled receptors with FAD as a cofactor

Per-Arnt-Sim (PAS; named after the representative proteins: Period, Aryl hydrocarbon receptor nuclear translocator protein, and Single-minded) domains stand as the most extensively prevalent sensing domains recognized in cytoplasmic chemoreceptors (Collins, Lacal & Ottemann, 2014; Stuffle, Johnson & Watts, 2021; Maschmann et al., 2022). These domains are capable of detecting a diverse spectrum of signals, encompassing oxygen, redox potential, blue light, and numerous other environmental stimuli (Taylor & Zhulin, 1999; Taylor, 2007; Stuffle, Johnson & Watts, 2021). Here, we focus on their role in oxygen-related signal sensing. *E. coli* Aer is one of the earliest and most well-understood aerotaxis receptors (Bibikov et al., 1997; Rebbapragada et al., 1997; Schweinitzer & Josenhans, 2010). The Aer monomer consists of a cytoplasmic N-terminal PAS domain, two transmembrane membrane-spanning segments, a cytoplasmic HAMP (histidine kinase, adenylyl cyclase, methyl-accepting chemotaxis protein, and phosphatase) domain, and a C-terminal coiled-coil kinase control domain (Amin, Taylor & Johnson, 2007; Maschmann et al., 2022). It functions in a dimeric form in *E. coli* cells and only responds to signals when anchored to the cell membrane (Greer-Phillips et al., 2003; Ma et al., 2004). Aer monitors intracellular redox potential of electron transport system (ETS) *via* its PAS domain, which noncovalently binds flavin adenine dinucleotide (FAD) (Bibikov et al., 1997; Bibikov et al., 2000; Garcia et al., 2016). FAD is indispensable for the sensory function of the receptor. The N-terminal segment (N-cap, residues 1–19) of the Aer PAS domain, along with the domain interior and adjacent regions, plays a critical role in FAD binding; amino acid mutations in these regions impair aerotaxis (Bibikov et al., 2000; Del Carmen Burón-Barral, Gosink & Parkinson, 2006; Watts et al., 2006a). The PAS domain and the HAMP domain within Aer form an input–output module for aerotaxis signal transduction through direct and lateral interactions (Fig. 1) (Watts et al., 2004; Ma, Johnson & Taylor, 2005; Del Carmen Burón-Barral, Gosink & Parkinson, 2006; Garcia et al., 2016). Missense mutations at certain amino acid sites within the HAMP domain also lead to abolished FAD binding to Aer (Watts et al., 2004; Ma, Johnson & Taylor, 2005). The signal transduction from the N-terminal PAS input domain to the C-terminal output domain occurs in a cis manner within one Aer monomer (Watts, Johnson & Taylor, 2006b). In addition to the involvement of Aer, the signal transduction process of aerotaxis in *E. coli* necessitates the collaborative participation of CheA, CheW, and CheY (Rowsell et al., 1995). When Aer is reconstituted into nanodiscs, it modulates the activity of the kinase CheA in a reversible manner, contingent upon the redox state of FAD within the higher-order complex comprising Aer, CheA, and CheW (Samanta et al., 2016). Specifically, the fully oxidized form of FAD enhances the kinase activity of CheA. In contrast, the reduction of oxidized FAD to its anionic semiquinone (ASQ) form results in the inhibition of CheA activity by Aer. These redox-induced changes in FAD prompt a conformational alteration in the PAS domain, which subsequently transmits the signal to the kinase control domain *via* the HAMP domain, thereby modulating the activity of CheA (Taylor, 2007; Samanta et al., 2016; Maschmann et al., 2022). This redox-dependent signaling mechanism underscores the pivotal role of FAD as a molecular switch in Aer-mediated aerotaxis. Furthermore, the mechanism by which FAD achieves oxidation or reduction in cells remains unclear (Maschmann et al., 2022).

AerC, derived from *A. brasilense*, is another representative PAS domain-coupled receptor that utilizes FAD as a cofactor. Unlike the membrane-bound Aer that harbors one PAS domain, AerC acts as a cytoplasmic receptor carrying two N-terminal PAS domains, enabling it to sense intracellular redox potential within a distinct spatial context (Greer-Phillips et al., 2003; Ma et al., 2004; Xie et al., 2010). FAD cofactors associate weakly with each of the two PAS domains of AerC *via* conserved tryptophan residues shared with Aer (Xie et al., 2010). Based on predicted topological and cellular localization differences, the two major conserved PAS domain subfamilies, typified by Aer and AerC, are designated as PAS_FAD1 and PAS_FAD2, respectively (Xie et al., 2010). PAS_FAD1 receptors are membrane-anchored, while the majority of PAS_FAD2 receptors are cytoplasmic soluble proteins (Xie et al., 2010). Although the precise mechanism by which AerC utilizes its two PAS domains to sense redox potential and transduce signals remains unclear, studies have shown that AerC mediates aerotaxis in *A. brasilense* and interacts with membrane-bound chemoreceptors to form signaling clusters at the pole under nitrogen-fixing conditions (Xie et al., 2010; Ganusova et al., 2023).

#### MCP-structured receptors with 4HB-type ligand-binding domain

*E. coli* encodes two aerotaxis receptors: Aer and the serine chemoreceptor Tsr, which exhibit distinct structural features in their N-terminal regions (Rebbapragada et al., 1997). The N-terminus of Aer contains a PAS domain that requires FAD as a cofactor. In contrast, the N-terminus of Tsr lacks a PAS domain and does not contain oxygen- or redox-responsive prosthetic groups (Greer-Phillips et al., 2003; Taylor, 2007). Tsr possesses a typical membrane-anchored chemoreceptor structure, with a periplasmic 4-helix-bundle (4HB)-type ligand - binding domain (LBD) at the N-terminus that can bind serine (Rebbapragada et al., 1997). As the second aerotaxis receptor in *E. coli*, Tsr serves a multifunctional chemoreceptor that responds to pH (Yang & Sourjik, 2012), temperature (Paulick et al., 2017), quorum signaling molecule autoinducer 2 (AI-2) (Hegde et al., 2011), blue light (Perlova et al., 2019) and others (Greer-Phillips et al., 2003). The aerotaxis receptors of *E. coli* detect changes in the electron transport system associated with variations in external oxygen concentration. Oxygen facilitates electron transport *via* the electron transport system, thereby enhancing the proton motive force (Laszlo & Taylor, 1981; Taylor, 1983). Tsr is capable of sensing the changes in proton motive force and modulating the movement of cells (Edwards, Johnson & Taylor, 2006). A larger jump in the proton motive force induces *E. coli* cells to exhibit a prolonged Tsr-mediated smooth-swimming response (Edwards, Johnson & Taylor, 2006). Analogous to the mechanism by which Tsr senses pH, it is plausible that Tsr’s perception of PMF may be mediated by charged amino acids within its HAMP domain (Umemura et al., 2002). Recently, AerA was identified as the primary aerotaxis transducer in *Pseudomonas syringae*, featuring a Tsr-like domain that supports the periplasmic 4HB-type ligand binding domain (Tumewu et al., 2022). It functions in conjunction with another protein, AerB, which contains a cytoplasmic PAS domain, to facilitate *P. syringae* aerotaxis (Tumewu et al., 2022; Ichinose et al., 2023). However, the mechanisms by which 4HB-type LBD-containing aerotaxis receptors perceive oxygen-related signals remain incompletely characterized, representing a key gap in current research.

### PilZ domain-coupled receptors

Specialized domains, such as the PilZ domain that binds the secondary messenger cyclic diguanylate monophosphate (c - di - GMP), are also found in some aerotaxis receptors (Amikam & Galperin, 2006; Russell et al., 2013; O’Neal et al., 2017; O’Neal, Akhter & Alexandre, 2019). C-di-GMP is a key signaling molecule regulating bacterial morphogenesis, developmental transitions, the motile-sessile switch, biofilm formation, virulence, and host–microbe symbiosis (Jenal, Reinders & Lori, 2017; Obeng et al., 2023). It also modulates the functional activity of specific chemoreceptors through allosteric regulation (Russell et al., 2013; O’Neal et al., 2017). *A. brasilense* encodes three aerotaxis receptors (AerC, Tlp1, Aer), with Tlp1 and Aer each containing a C-terminal PilZ domain (Greer-Phillips, Stephens & Alexandre, 2004; Xie et al., 2010; O’Neal, Akhter & Alexandre, 2019). Oxygen is the primary factor regulating the concentration of c-di-GMP (Russell et al., 2013). Fluctuations in the intracellular oxygen concentration can result in swift variations in the intracellular levels of c-di-GMP (O’Neal et al., 2017). Although Tlp1 lacks a typical N-terminal oxygen-sensing domain, c-di-GMP binding to its PilZ domain modulates receptor sensitivity to oxygen gradients (Greer-Phillips, Stephens & Alexandre, 2004; Russell et al., 2013; O’Neal et al., 2017). Gene knockout and phenotypic analyses have demonstrated that the PilZ domain of *A. brasilense* Aer is critical for the aerotactic response but has no effect on organic acid chemotaxis (O’Neal, Akhter & Alexandre, 2019). In addition to the PilZ domain, *A. brasilense* Aer shares a similar topological structure and domain organization with *E. coli* Aer, and its N-terminal PAS domain is suggested to bind FAD noncovalently, which may contribute to oxygen signal transduction alongside c-di-GMP-mediated regulation (O’Neal, Akhter & Alexandre, 2019).

### Bipartite receptors

Within the extensive array of receptors, bipartite receptors represent a distinctive class found in various bacterial genomes, characterized by their formation through the interaction of two proteins (Elliott & Di Rita, 2008; Reuter & Van Vliet, 2013; Herz et al., 2025). A bipartite receptor involved in energy taxis has been identified in *Campylobacter jejuni* (Elliott & Di Rita, 2008; Elliott et al., 2009). In *C. jejuni*, this receptor is composed of two distinct proteins: an integral membrane protein named CetA, which features a transmembrane region, a HAMP domain, and a highly conserved signaling domain (HCD), and a peripheral membrane protein referred to as CetB that contains an isolated PAS domain (Fig. 1) (Elliott & Di Rita, 2008; Elliott et al., 2009). The *cetA* and *cetB* genes are co-transcribed in the genome of *C. jejuni*, with the stability of CetB being reliant on CetA, and the two proteins forming larger complexes to facilitate signaling (Elliott & Di Rita, 2008). Here, CetB binds FAD to sense redox changes, while CetA is responsible for signal transduction. The CetA/CetB complex is homologous to chemoreceptors in other species, where signal input is mediated through PAS domains containing FAD, akin to the Aer in *E. coli*, indicating conserved mechanisms for energy sensing (Elliott et al., 2009; Xie et al., 2010). The primary distinction is that CetA is predicted to possess a canonical HAMP domain characterized by two amphipathic helices, whereas the HAMP domain in *E. coli* comprises one amphipathic helix and one hydrophobic helix (Ma, Johnson & Taylor, 2005; Elliott et al., 2009). This finding suggests that the signal transduction function of the HAMP domain in CetA diverges from that of the HAMP domain in Aer (Elliott et al., 2009). Furthermore, the soluble protein CetZ (Tlp8), which contains two PAS domains and one MCP domain, acts antagonistically to the CetA/CetB system by counterbalancing its signaling output, thereby directing *C. jejuni* cells toward optimal energy resources (Reuter & Van Vliet, 2013). Importantly, the bipartite receptor architecture is conserved. In the model magnetotactic bacterium *Magnetospirillum gryphiswaldense*, the bipartite receptor CetA_2_/CetB_2_ mediates an oxygen repellent response through CetB2’s FAD-dependent redox sensing (Herz et al., 2025). Analogous to the mechanism observed in *C. jejuni*, it is proposed that CetB2 transmits signals through interaction with CetA2, thereby altering flagellar movement to direct cells toward optimal oxygen concentrations or suitable niches for energy metabolism (Elliott & Di Rita, 2008; Elliott et al., 2009; Herz et al., 2025). This discovery represents the first elucidation of the molecular mechanism underlying oxygen sensing regulation in magnetotactic bacteria. Collectively, these studies indicate that bipartite chemoreceptor complexes, characterized by PAS domain-containing subunits and FAD-dependent redox sensing, constitute a conserved strategy for aerotaxis regulation across diverse bacterial species.

### Direct oxygen-sensing aerotaxis or aerotaxis-like receptors

#### PAS domain-coupled receptors with heme as a cofactor

PAS domains are multifunctional structural modules. Some of which bind a heme cofactor rather than FAD, enabling the bacterium to respond to oxygen concentrations directly. IcpB of *Azorhizobium caulinodans* possesses a heme-binding PAS domain at its N-terminus and is characterized as a cytoplasmic aerotaxis receptor with a relatively simple domain composition (Jiang et al., 2016). However, another group of aerotaxis-like receptors represented by Aer2 containing such PAS domains generally exhibit more complex domain architectures than IcpB (Garcia et al., 2017; Greer-Phillips et al., 2018; Orillard & Watts, 2022; Stuffle et al., 2022). Although the roles of these receptors in aerotaxis remain controversial or unproven, their capacity to sense oxygen has been well confirmed (Hong et al., 2004a; Garcia et al., 2017; Orillard & Watts, 2022). As a typical example, *Pseudomonas aeruginosa* Aer2 carries a PAS domain that coordinates b-type heme *via* histidine residue H234; while its role in native aerotaxis is debated, heterologous expression of Aer2 in *E. coli* mediates repellent tumbling (signal-on) responses to O_2_ (Hong et al., 2004a; Watts, Taylor & Johnson, 2011; Garcia et al., 2017). The PAS domain of Aer 2 is flanked by three N-terminal and two C-terminal HAMP domains, designated HAMP1 to HAMP5 in sequence (Garcia et al., 2017). Unlike *E. coli* Aer, which achieves signal transduction through the interaction between the PAS domain and the HAMP domain, the interaction between the PAS domain and the HAMP domain in Aer2 appears to be minimal and all domains of Aer2 are arranged linearly (Ma, Johnson & Taylor, 2005; Del Carmen Burón-Barral, Gosink & Parkinson, 2006; Airola et al., 2013; Garcia et al., 2016). This structural composition of domains renders its signal transduction quite distinctive. HAMP1, which is separated from HAMP2-3 by a helical extension, is largely dispensable for Aer2 function (Watts, Taylor & Johnson, 2011; Airola et al., 2013). In contrast, HAMP2-3 plays a crucial role in stabilizing the PAS signaling state by altering its conformation in response to PAS ligand binding (Watts, Taylor & Johnson, 2011; Garcia et al., 2017). The C-terminal HAMP4-5 jointly function as a unit to affect the kinase control module (Watts, Taylor & Johnson, 2011). Based on current experimental evidence, the current signaling model suggests that when the PAS domain binds to the ligand, the conformational signal from the PAS domain is transmitted to the C-terminal HAMP4-5, HAMP4 and HAMP5 no longer inhibit the kinase control module, ultimately leading to the autophosphorylation of the downstream histidine kinase CheA2 (Watts, Taylor & Johnson, 2011; Garcia et al., 2017). The conformational changes induced in the PAS domain upon ligand binding may be directly transmitted to the downstream HAMP4 and HAMP5, as a conserved motif connecting the PAS domain and the C-terminal HAMP domains is crucial for signal transduction (Airola et al., 2013; Garcia et al., 2017; Orillard et al., 2021).

Aer2 receptors have also been identified in other bacterial species, including *Vibrio cholerae* (Greer-Phillips et al., 2018), *Leptospira interrogans* (Orillard & Watts, 2022), and *Vibrio vulnificus* (Stuffle et al., 2022). These Aer2 orthologs share structural features with *P. aeruginosa* Aer2, binding heme *via* conserved histidine residues and mediating oxygen sensing in *E. coli* (Greer-Phillips et al., 2018; Orillard & Watts, 2022; Stuffle et al., 2022). However, they exhibit notable differences in the number of PAS and HAMP domains compared to *P. aeruginosa* Aer2: *V. cholerae* Aer2 contains two N-terminal PAS domains, while *V. vulnificus* and *L. interrogans* Aer2 encode three N-terminal PAS domains (Greer-Phillips et al., 2018; Stuffle et al., 2022; Orillard & Watts, 2022). In *V. vulnificus* Aer2, all three PAS domains bound heme and demonstrated similar O_2_ affinities (Stuffle et al., 2022). However, their roles in signal transduction differ. PAS1 is regarded as largely indispensable for oxygen-mediated signal transduction, while PAS2 regulates the signal transduction of PAS3, which transmits signals to downstream domains (Stuffle et al., 2022). *L. interrogans* Aer2 was found to induce *E. coli* cells to tumble in the absence of oxygen, whereas the presence of oxygen inhibits *E. coli* tumbling (Orillard & Watts, 2022). This outcome is contrary to the function of *P. aeruginosa* Aer2 in *E. coli* (Watts, Taylor & Johnson, 2011). The reason for this requires further investigation. These studies collectively validate Aer2’s function as an oxygen sensor in diverse bacterial species. Moreover, they uncover distinct signaling responses and transmission mechanisms resulting from the diverse combinations of its N-terminal domains. This offers critical perspectives for subsequent research undertakings.

#### Globin domain-coupled receptors with heme as a cofactor

Globin-coupled sensors (GCS) are heme-containing proteins found in bacteria, archaea, and lower eukaryotes (Hoque & Weinert, 2023). They belong to a larger family of heme-based sensors that respond to changes in oxygen level, playing critical roles in regulating O_2_-dependent physiology and phenotypes (Walker, Rivera & Weinert, 2017). GCS consists of an N-terminal sensor globin domain linked to various C-terminal output domains (Hou et al., 2001a). Based on the structural variations in the C-terminal output domain, putative GCS include histidine kinases, diguanylate cyclases (DGC), adenylate cyclases (AC), methyl-accepting chemotaxis proteins, and phosphodiesterases (Walker, Rivera & Weinert, 2017; Hoque & Weinert, 2023; Kitanishi, Aoyama & Shimonaka, 2024). Aerotaxis receptors characterized by a globin domain at the N-terminus and a signaling domain homologous to the cytoplasmic signaling domains of bacterial chemoreceptors at the C-terminus are classified as members of the GCS family (Hou et al., 2001a). To date, experimentally validated GCS family members regulating aerotaxis include HemAT from *Bacillus subtilis* and *Halobacterium salinarium* (Hou et al., 2000; Hou et al., 2001a), GCS_*Bh*_ from *Bacillus halodurans* C-125 (Hou et al., 2001b), and Atu1027 from *Agrobacterium tumefaciens* (Huang et al., 2024).

HemAT-Bs (derived from *B. subtilis*), the initially identified aerotaxis receptor, is composed of a globin domain, a signaling domain, and a linker region that connects these two (Hou et al., 2000; Ohta et al., 2004; El-Mashtoly et al., 2008). It reversibly binds diatomic oxygen *via* a heme cofactor located in its N-terminal globin domain, a process accompanied by conformational changes (Hou et al., 2001b; Freitas et al., 2005; El-Mashtoly et al., 2008). The globin domain of HemAT-Bs can not only bind O_2_, but also reversibly bind gas molecules such as CO, NO (Liu & Kincaid, 2021). The conformational changes that occur within the globin domain when binding different gas molecules can be distinguished through subtle differences in heme-pocket dynamics and ligand-binding kinetics (Yoshimura et al., 2006; Yoshida et al., 2012; Liu & Kincaid, 2021). Here, we focus exclusively on conformational changes induced by oxygen binding. The signaling domain of HemAT shares a similar protein architecture with the cytoplasmic domains of other chemoreceptors (Zhang, Olson & Phillips, 2005). Full-length HemAT-Bs forms dimers with an elongated, asymmetric morphology, whereas their structure becomes more symmetrical upon ligand binding (Zhang & Phillips, 2003). This symmetry disruption in HemAT-Bs is caused by a unique conformational change in the one subunit of the dimer (Zhang, Olson & Phillips, 2005). The conformational change is subsequently propagated through the middle region to control interactions of the C-terminal signaling domains, which triggers a downstream signaling cascade (Freitas et al., 2005; Yoshida et al., 2012; Walker, Rivera & Weinert, 2017). Specifically, HemAT-Bs interacts with the downstream histidine kinase CheA, transmitting signals to regulatory proteins that control flagellar rotation direction (Hou et al., 2000). Due to the presence of components with both high and low affinity to oxygen, HemAT is capable of reacting to gradients in oxygen concentration in both hypoxic and aerobic environments (Zhang, Olson & Phillips, 2005). This characteristic could potentially be significant for an effective sensing system.

The globin domain of HemAT-Bs features a distinctive distal heme pocket, which is surrounded by Tyr70, Thr95, and a water molecule (Zhang & Phillips, 2003). In the heme pocket, His123 serves as the proximal residue for heme binding in HemAT-Bs (Hou et al., 2001a). Tyr70 plays a crucial role in signal transduction, as an obvious conformational change occurs in Tyr70 upon the removal of ligands, which may be critical for the signaling transduction process (Zhang & Phillips, 2003; Ohta et al., 2004; Martínková, Kitanishi & Shimizu, 2013). The side chain of Tyr70, which exhibits highly dynamic behavior, is strongly influenced by the nature of the iron-ligand complex (Zhang, Olson & Phillips, 2005). Thr95 is considered the key residue responsible for sensing oxygen bound to heme, under the premise that His86 forms hydrogen bonds with the heme propionate in the O_2_-bound form, Thr95 can form hydrogen bonds with the heme-bound O_2_ (Ohta et al., 2004; Yoshimura et al., 2006; El-Mashtoly et al., 2008). This complex hydrogen-bonding network, with Tyr70 and Thr95 at its core, regulates allosteric communication between the heme pocket and distal signaling domains, which is the molecular basis for the precise transmission of oxygen binding signals to the downstream chemotaxis system.

Research on HemAT-Hs (from *H. salinarium*) and GCS_*Bh*_ is relatively limited, the characterization studies indicate that their behavioral patterns are similar to those of HemAT - Bs (Walker, Rivera & Weinert, 2017). The newly identified Atu1027 protein from *A. tumefaciens* may respond to different oxygen concentrations *via* a similar mechanism, although direct evidence for heme binding by its globin domain is lacking (Huang et al., 2024). This inference is supported by the evidence of site-directed mutagenesis targeting a predicted essential histidine residue at position 100 (His100), which is anticipated to play a key role in heme binding (Hou et al., 2001a; Huang et al., 2024).

### Specialized domain-coupled receptors

The non-heme iron oxygen-carrying protein hemerythrin was initially identified in certain marine invertebrates. Subsequently, a methyl-accepting chemotaxis protein, DcrH, which features a hemerythrin-like domain at its C-terminus, was discovered in the anaerobic sulfate-reducing bacterium *Desulfovibrio vulgaris* (Xiong et al., 2000). The complete structure of DcrH consists of a periplasmic membrane domain, a HAMP domain, an MCP signal domain, and a hemerythrin-like domain (Kitanishi, 2022). The hemerythrin-like domain of DcrH contains a diiron site that binds oxygen (Xiong et al., 2000). Upon binding oxygen, the hemerythrin-like domain undergoes redox-dependent conformational changes, which may propagate to the adjacent methylation domain (Isaza et al., 2006). Consequently, DcrH is hypothesized to enable the bacterium to exhibit negative aerotaxis (anaerotaxis). However, the mechanism of signal transduction from the hemerythrin-like domain to the MCP domain in the full-length protein remains inadequately understood, which limits our understanding of how anaerobic bacteria adapt to extreme environments through oxygen sensing (Isaza et al., 2006; Kitanishi, 2022).

In addition to the hemerythrin-like aerotaxis receptor found in bacteria, archaea also possess structurally unique aerotaxis receptors. HtrVIII, identified in the archaeon *H. salinarium*, displays a distinctive structure characterized by an N-terminal region composed of six transmembrane segments and a C-terminal domain that shows significant homology with the eubacterial methyl-accepting chemotaxis protein (Brooun et al., 1998). The six transmembrane segments, homologous to the heme-binding sites in eukaryotic cytochrome *c* oxidase, likely bind heme to directly sense oxygen concentration (Brooun et al., 1998). Notably, HtrVIII is the first archaeal MCP reported to mediate aerophilic responses, in contrast to HemAT-Hs, which triggers aerophobic behavior (Brooun et al., 1998; Hou et al., 2000).

### Structural classification and organizational patterns of aerotaxis receptors

The differential specialization of receptors is fundamentally determined by their structural characteristics, including ligand-binding domain (LBD) type and membrane topology. Chemoreceptors are generally classified into four classes (I–IV) based on these features (Lacal et al., 2010). The main characteristics of these four classes are as follows: Class I contains one or two transmembrane helices linked to a periplasmic LBD; Class II lacks extracellular structures but has two transmembrane helices, with one end connected to an intracellular LBD; Class III has a variable number of transmembrane helices but no periplasmic LBD; and Class IV is entirely intracellular, lacking transmembrane helices (Lacal et al., 2010; Salah Ud-Din & Roujeinikova, 2017). Aerotaxis receptors have been identified across all four classes, with Class II (membrane-bound) and Class IV (cytoplasmic) being the most prevalent (Table S1). Understanding the structural organization of these receptors in the chemotaxis system provides critical insights into their aerotactic functions. In the *E. coli* chemotaxis system, membrane-bound chemoreceptors form trimer-of-dimers that assemble into large polar sensory arrays (Cassidy et al., 2023; Liu et al., 2025). These trimers of chemoreceptor dimers are coupled with cytoplasmic proteins to form interconnected hexagonal signaling units, whose cytoplasmic tips interact with rings of CheA and CheW to form a cytoplasmic signaling baseplate (Parkinson, Hazelbauer & Falke, 2015; Liu et al., 2025). Aer, similar to other *E. coli* chemoreceptors (such as Tar and Tsr), assembles into higher-order signaling complexes by interacting with CheA and CheW, although direct evidence of Aer receptor clustering has not yet been observed in cells (Samanta et al., 2016). As far as we know, apart from *E. coli* Aer, there have been no reports on the study of other membrane-bound aerotaxis receptors in forming large polar sensory arrays.

Studies on cytoplasmic receptors have revealed unique subcellular localization and functional characteristics. In *B. subtilis,* HemAT-Bs aggregates with transmembrane chemoreceptors at the poles, implying that it may function in conjunction with other chemoreceptors (Cannistraro et al., 2011). Cytoplasmic receptors lack structures anchored to the cell membrane, which may lead to unstable localization within the cell. However, this may not be the case. Some cytoplasmic receptors can form stable signaling clusters with membrane-bound chemoreceptors at the cell pole, thus ensuring the stability of their subcellular localization and the efficiency of signal transduction (Güvener, Tifrea & Harwood, 2006; Ganusova et al., 2023). In the early stable phase of *P. aeruginosa* growth, multiple proteins of the Che2 chemosensory system cluster at the cell pole, and this clustering is maintained solely by the predicted cytoplasmic receptor Aer2 (Güvener, Tifrea & Harwood, 2006). Notably, Che2 proteins organized by Aer2 do not share the same location as the Che proteins of other chemotaxis system (Güvener, Tifrea & Harwood, 2006). This spatial segregation implies functional specialization, in which Aer2-mediated Che2 clustering allows it to operate without interference from other chemotactic pathways. This finding also highlights the critical role of cytoplasmic chemoreceptors in organizing chemotaxis proteins. A study on *A. brasilense* AerC and Tlp4b demonstrated that deletion of cytoplasmic receptor AerC or Tlp4b leads to a decrease in the polar localization of membrane-bound chemoreceptors at the cell poles and disrupts the organization of chemotaxis signaling clusters (Ganusova et al., 2023). Notably, AerC dynamically traffics into and out of chemotaxis signaling clusters in an expression level-dependent manner, while Tlp4b, which is capable of participating in chemotaxis and wheat root surface colonization, does not exhibit this behavior (Ganusova et al., 2023). Previous studies have shown that overexpression of aerotaxis receptors exhibit stronger redox taxis (Rebbapragada et al., 1997; Xie et al., 2010) or aerotaxis (Brooun et al., 1998; Boin & Hase, 2007; Huang et al., 2024). Under nitrogen-fixing conditions, the expression level of AerC was precisely upregulated (Xie et al., 2010). Thus, cytoplasmic receptors such as *A. brasilense* AerC and *P. aeruginosa* Aer2 may mediate refined regulation of signal perception through their role in organizing polar chemotaxis signaling clusters (Güvener, Tifrea & Harwood, 2006; Ganusova et al., 2023).

### Differential specialization of aerotaxis receptors and microbial adaptive flexibility

The core mechanism underlying microbial adaptive flexibility to complex environmental changes is the differential specialization of multiple aerotaxis receptors within single strains or across different species. These specialized receptors cooperate or antagonize to modulate aerotaxis behavior, enabling bacteria to precisely locate optimal oxygen niches. Current experimental studies and bioinformatics analyses, aerotaxis receptors are widely distributed in aerobic, microaerophilic, and even anaerobic bacteria (Fu, Wall & Voordouw, 1994; Hou et al., 2000; Alexandre, 2010; Schweinitzer & Josenhans, 2010; Xie et al., 2010; Walker, Rivera & Weinert, 2017). Within the same bacterial species, receptor quantities range from one to multiple. These different types of receptors endow microorganisms with unique abilities to either move towards suitable oxygen conditions or avoid aerobic environments, allowing them to adapt to the ever-changing external environment and to modulate the interactions with the host. When two or more receptors mediate positive aerotaxis, structural differences lead to distinct response mechanisms or functional specialization. In *E. coli*, Aer and Tsr act synergistically to detect oxygen gradients: Aer responds to rapid changes in intracellular energy status, while Tsr mediates sustained responses to PMF changes, enabling *E. coli* to precisely locate optimal oxygen concentrations (Rebbapragada et al., 1997; Edwards, Johnson & Taylor, 2006). Although both are energy receptors, the signals they perceive are markedly different. This sensing mechanism may enable *E. coli* to promptly detect rapid changes in environmental factors and respond accordingly. Another example is the three aerotaxis receptors of *A. brasilense* (AerC, Tlp1, and Aer). Among them, Tlp1 and Aer possess a unique PilZ domain at their C-terminus, which can bind the intracellular second messenger c-di-GMP (Russell et al., 2013; O’Neal et al., 2017; O’Neal, Akhter & Alexandre, 2019). The levels of c-di-GMP can regulate the ability of cells to detect optimal oxygen concentration in a spatial gradient of air, ultimately influencing the aerotaxis behavior of *A. brasilense* (Russell et al., 2013; O’Neal et al., 2017). Tlp1 and Aer are also required for plant root colonization, with the PilZ domain playing a critical role in this process (Greer-Phillips, Stephens & Alexandre, 2004; O’Neal, Akhter & Alexandre, 2019). In contrast, no analogous mechanism has been identified in AerC, which serves as the principal aerotaxis receptor of *A. brasilense* under nitrogen—fixing conditions (Xie et al., 2010; O’Neal, Akhter & Alexandre, 2019). For *P. syringae* AerA and AerB, the molecular mechanisms of aerotaxis remain unclear, but structural differences suggest that they may mediate aerotaxis *via* distinct pathways (Tumewu et al., 2022). Notably, not all aerotaxis receptors drive positive aerotaxis; some mediate negative aerotaxis, as exemplified by *H. salinarium*. HemAT-Hs and HtrVIII play opposing roles in aerotaxis regulation. HemAT-Hs is essential for the aerophobic response (Hou et al., 2000). HtrVIII mediates aerophilic responses (Brooun et al., 1998). In wild-type *H. salinarium* cells, the aerophobic response is partially masked by the strong aerophilic response when both receptors are present (Hou et al., 2000). In anaerobic bacteria *D. vulgaris*, DcrH is also a special receptor that directly binds oxygen and triggers oxygen avoidance behavior, which may prevent oxidative damage to anaerobic metabolic systems (Isaza et al., 2006). Based on the currently limited research, how these receptors mediate negative aerotaxis remains unclear.

### Adaptation in different aerotaxis receptors

Oxygen acts as either an attractant or a repellent for bacteria, depending on their aerobic metabolic characteristics and the ambient oxygen concentration (Shioi, Dang & Taylor, 1987; Grishanin & Bibikov, 1997). Aerobic and facultative anaerobic species exhibit positive aerotaxis, while anaerobic species show negative aerotaxis (Armitage, 1997; Taylor, Zhulin & Johnson, 1999). Beyond microbial aerobic requirements, oxygen concentration modulates its role in aerotaxis: high oxygen levels act as a repellent for *E. coli*, *S. typhimurium*, and certain bacilli, while low oxygen levels serve as an attractant (Shioi, Dang & Taylor, 1987). Diverse aerotaxis receptors enable bacteria to detect oxygen gradients *via* direct or energy-dependent indirect mechanisms, and the persistence of oxygen signals in the environment has driven the evolution of adaptive mechanisms to avoid signal desensitization. In the chemotaxis system, the best-understood mechanism of adaptation relies on the methylation and demethylation of chemoreceptors, processes catalysed by the methyltransferase CheR and the methylesterase CheB (Collins, Lacal & Ottemann, 2014; Tran et al., 2023). This mechanism enables bacterial swimming behavior to revert to the frequency of directional changes observed prior to the stimulation by attractants after undergoing a phase of smooth swimming induced by these attractants.

In certain bacteria, such as *H. salinarium* (Lindbeck et al., 1995; Brooun et al., 1998), *P. aeruginosa* (García-Fontana, Corral Lugo & Krell, 2014; Sheng et al., 2019), and *B. subtilis* (Wong et al., 1995), adaptation is also dependent on methylation and demethylation. In *H. salinarium*, HemAT-Hs undergoes methylation by CheR *in vivo*, which enhances the activation of CheA and the downstream aerotactic signaling (Lindbeck et al., 1995; Hou et al., 2000; Schlesner et al., 2012). Methylation of *P. aeruginosa* Aer is catalyzed by CheR1 (Sheng et al., 2019). The *P. aeruginosa* Aer2 (McpB) protein is also expected to undergo fine-tuning through demethylation and methylation processes, which are regulated by CheB2 and CheR2, respectively (García-Fontana, Corral Lugo & Krell, 2014; Garcia et al., 2017). However, when Aer2 is expressed in chemoreceptor-less *E. coli*, it induces continuous tumbling of the cells, resulting in an oxygen-repellent response (Watts, Taylor & Johnson, 2011). This process is independent of Aer2 methylation (Garcia et al., 2017). Curiously, although methylation plays a role in the aerotaxis of *B. subtilis*, no methylation changes have been detected in its aerotaxis receptor HemAT-Bs to date (Wong et al., 1995; Walker, Rivera & Weinert, 2017). This paradox was partially resolved by the discovery of monotonic aerotaxis. *B. subtilis* exhibits a robust aerotactic behavior but lacks the anaerobic avoidance response to hyperoxia characteristic of *E. coli* and other enteric bacteria (Wong et al., 1995). Subsequent study has revealed that *B. subtilis* exhibits continuous migration towards the maximum oxygen concentration (monotonic aerotaxis) in microfluidic devices capable of precisely controlling oxygen concentration gradients across the entire range from near-anoxia to hyperoxia (60 nmol/Lto1 mmol/L), thus resolving the ‘oxygen preference conundrum’ (Menolascina et al., 2017). Thus, the aerotaxis of *B. subtilis* and the potential role of other methylatable aerotaxis receptors require further investigation. At the same time, some bacteria also exhibit methylation-independent adaptation. The most typical examples are the adaptations observed in *E. coli* and *S. typhimurium* (Taylor, 1983; Dang et al., 1986). Although *E. coli* Aer contains three putative methylation sites in its C-terminus, strains lacking CheR or CheB, or having site-specific mutations at potentially methylatable sites within the signaling domain of Aer, are still capable of exhibiting aerotaxis (Rebbapragada et al., 1997; Bibikov et al., 2004). Even when the PAS - HAMP domain of Aer is fused with the signaling domain of Tar (a methylation- dependent chemoreceptor in *E. coli*), aerotaxis signaling remains methylation-independent in strains deficient in both CheR and CheB (Bibikov et al., 2004). These observations collectively indicate that methylation-independent adaptation in Aer is mediated by the PAS-HAMP domain. This adaptation of Aer potentially transpires through transient modifications in the redox state of the FAD cofactor non-covalently linked to the PAS domain of Aer (Alexandre, 2010). Subsequent studies have provided experimental evidence to support this hypothesis. Upon FAD oxidation to its fully oxidized state (FAD_OX_), Aer activates CheA kinase activity, whereas FAD_OX_ reduction to the anionic semiquinone state (FAD_ASQ_) suppresses CheA activity, with such kinase modulation may ultimately switch the motility of *E. coli* from tumbling to smooth swimming (Samanta et al., 2016; Maschmann et al., 2022). Whether such a switch in the motility pattern of *E. coli* indeed occurs following a shift from high to low ambient oxygen concentrations remains to be verified by further experimental evidence.

In summary, both receptor-dependent methylation and non-methylation regulation have been observed in aerotaxis (Bibikov et al., 2004; Stephens, Loar & Alexandre, 2006). Given that not all aerotaxis-associated receptors are methylated, the collective term aerotaxis receptors is more appropriate for these proteins.

### Ecological roles of aerotaxis

The structural diversity and specific organizational patterns of aerotaxis receptors lay the molecular foundation for the precise perception and transduction of oxygen signals, and this well-regulated aerotaxis response further mediates a variety of ecological processes of bacteria in natural environments.

#### Aerotaxis contributes to the interaction between bacteria and host

Aerotaxis assumes a crucial function in mediating host-microbe interactions, especially within pathogenic and symbiotic associations. It empowers bacteria to traverse intricate environments, identify optimal survival niches, and initiate infection or colonization procedures. For bacterial pathogens, aerotaxis is a prerequisite for effective host infection. The opportunistic human pathogen *P. aeruginosa*, which infects diverse plant and animal hosts, exhibits reduced virulence across infection models when its aerotaxis-like receptor Aer2 is deleted, highlighting Aer2’s regulatory role in virulence (García-Fontana et al., 2019). Similar deficiencies are observed in phytopathogens like *R. solanacearum*, which relies on aerotaxis transducers for oxygen gradient detection. Mutants without these transducers show delayed disease onset in tomatoes and impaired root localization, emphasizing aerotaxis’s importance in host colonization (Yao & Allen, 2007). Analogous findings have been reported in *P. syringae* and *A. tumefaciens*, where deletion of aerotaxis receptors diminishes bacterial pathogenicity on plant parts (Tumewu et al., 2022; Ichinose et al., 2023; Huang et al., 2024). Aerotaxis is likely instrumental in facilitating host colonization by guiding pathogens to essential entry sites. For instance, the foliar pathogen *P. syringae* is postulated to utilize oxygen emitted from leaf stomata to identify infection sites, thereby enabling leaf colonization and symptom manifestation (Ichinose et al., 2023). In *A. tumefaciens*, which colonizes hypoxic crown gall tumors at wound sites, a pronounced ability to seek oxygen may enhance rapid replication and persistent infection by targeting optimal oxygen microenvironments (Gohlke & Deeken, 2014; Huang et al., 2024). Beyond pathogenic interactions, aerotaxis also plays a role in symbiotic and biocontrol processes. In the context of biological control, *P. chlororaphis* PCL1606 utilizes aerotaxis for avocado root colonization and suppression of the pathogen *Rosellinia necatrix* (Arrebola & Cazorla, 2020). In symbiotic relationships, IcpB in *A. caulinodans* is involved in sensing oxygen gradients and organic acid chemotaxis; disruption of this receptor impairs extracellular polysaccharide production, flocculation, and competitive nodulation with the legume host *Sesbania rostrata* (Jiang et al., 2016). Similarly, in *A. brasilense*, the aerotaxis receptors AerC, Tlp1, and Aer are collectively essential for colonization of wheat roots, with the deletion of AerC completely abolishing this capability (Greer-Phillips, Stephens & Alexandre, 2004; O’Neal, Akhter & Alexandre, 2019; Ganusova et al., 2023).

The interactions between bacteria and host are notably complex. While aerotaxis positively regulates these processes from an outcome perspective, the roles of aerotaxis receptors are likely multifaceted. On one hand, the diversification of signal recognition suggests that receptors classified under energy taxis can detect signals not only from oxygen but also from oxidizable substrates, pH variations, and other factors (Alexandre, 2010; Schweinitzer & Josenhans, 2010). Some receptors can bind to the second messenger c-di-GMP through specialized domains, thereby influencing the symbiosis between bacteria and plants (Amikam & Galperin, 2006; Russell et al., 2013; O’Neal et al., 2017). C-di-GMP is an important signaling molecule that can affect virulence and host–microbe symbiosis (Jenal, Reinders & Lori, 2017; Obeng et al., 2023). This diversified mechanism of signal recognition may facilitate a more rapid response to various environmental signal changes during host interactions. On the other hand, both chemotaxis and aerotaxis are critical for the interactions between bacteria and their hosts (Matilla & Krell, 2018; Raina et al., 2019). Aerotaxis receptors may influence the stability and functionality of large chemosensory arrays and the efficiency of signal transduction in chemotaxis (Güvener, Tifrea & Harwood, 2006; Ganusova et al., 2023). Additionally, the influence of gene expression regulation may also be present. In the marine animal pathogen *Vibrio alginolyticus* (ND-01), silencing the *aer* gene results in significant downregulation of genes related to *cheA*, *cheW*, and *cheY*, leading to deficiencies in adhesion, chemotaxis, flagellar assembly, and motility in the mutant strains (Huang et al., 2017).

### Impact of aerotaxis on biofilm formation

Bacteria have the ability to form biofilms on solid surfaces or at the air-liquid interface, with aerotaxis playing a significant role in this process (Hölscher et al., 2015; Wu & Jin, 2019). This phenomenon is particularly pronounced in various bacterial species. However, the involvement of aerotaxis receptors in biofilm formation remains a contentious issue. Some aerotaxis receptors function as negative regulators of biofilm formation, and impaired aerotaxis receptors have been shown to enhance biofilm formation in bacteria (Yao & Allen, 2007; Jiang et al., 2016). In the case of the microaerophilic *R. solanacearum*, this enhancement may be attributed to a deficiency in aerotaxis receptors, which results in a reduced ability to evade the toxic effects of high oxygen levels, thereby prompting the adoption of a protective strategy (Yao & Allen, 2007). In the obligate aerobe *A. caulinodans*, biofilm formation may be augmented through certain unknown mechanisms (Jiang et al., 2016). In contrast, aerotaxis and aerotaxis receptors in various bacterial species play a pivotal role in facilitating biofilm formation (Armitano, Méjean & Jourlin-Castelli, 2013; Hölscher et al., 2015; Arrebola & Cazorla, 2020; Shu et al., 2022; Tumewu et al., 2022; Huang et al., 2024). For instance, in *B. subtilis,* biofilm formation at air-liquid interfaces is significantly influenced by motility, chemotaxis and aerotaxis, which are crucial for maintaining competitive fitness during biofilm development (Hölscher et al., 2015). The relationship between aerotaxis and biofilm formation is further highlighted by findings in *C. jejuni*, where flagellar motility and chemotaxis are essential for the structured development of biofilms (Reuter et al., 2020). Importantly, aerotaxis is intricately linked to bacterial flagellar motility and chemotaxis; any disruption in aerotaxis receptors can lead to impaired motility and chemotaxis, consequently affecting the spatial colonization of bacteria necessary for effective biofilm formation (Xie et al., 2010; Arrebola & Cazorla, 2020; Tumewu et al., 2022; Huang et al., 2024). Additionally, research has demonstrated that in *Comamonas testosteroni* CNB-1, specific chemoreceptors physically interact with components that are integral to the biofilm formation pathway (Huang et al., 2019b). The histidine kinase CheA has been observed to phosphorylate FlmD, a response regulator that acts as a key factor in mediating biofilm formation (Huang et al., 2019b). Therefore, investigating the potential crosstalk among various signaling systems may enhance our understanding of the relationship between aerotaxis and biofilm formation. Moreover, oxygen-sensor interactions can modulate the activity of downstream signaling pathways, such as those involving diguanylate cyclase, which is vital for biofilm formation in bacteria (Burns et al., 2016).

## Conclusions and perspectives

Aerotaxis constitutes a vital survival mechanism that facilitates motile bacteria and archaea in orienting themselves towards optimal oxygen concentrations necessary for metabolism and growth. This process is orchestrated by aerotaxis receptors, which detect oxygen or related signals and transmit this information to the chemotaxis systems, ultimately directing the flagellar movement of cells. Typically, these receptors are characterized by the presence of PAS or globin domains, although some also incorporate specialized functional domains such as PilZ or hemerythrin-like domains, and may even manifest as bipartite receptors (Hou et al., 2000; Elliott & Di Rita, 2008; Alexandre, 2010; Xie et al., 2010; Walker, Rivera & Weinert, 2017; O’Neal, Akhter & Alexandre, 2019; Kitanishi, 2022; Herz et al., 2025). The diversity of aerotaxis receptors reflects the varied ecological niches and metabolic strategies of different motile prokaryotes. Remarkably, in the non-flagellated bacterium *Corallococcus coralloides*, a unique aerotaxis methyl-accepting chemotaxis protein (MCP) has been predicted through *in silico* analyses within a putative energy taxis cluster (Sharma, Parales & Singer, 2018). This MCP is characterized by the presence of both a PAS domain and a globin domain, and it is hypothesized to confer an advantage in oxygen sensing, either directly or indirectly (Sharma, Parales & Singer, 2018). This discovery offers novel perspectives for comprehending the mechanisms through which these myxobacteria sense and efficiently respond to alterations in oxygen concentration.

Recent investigations into aerotaxis receptors have predominantly concentrated on elucidating their molecular mechanisms. Despite the identification of several receptors, the extent to which some contribute to the regulation of aerotaxis remains ambiguous. This uncertainty is particularly pronounced in strains harboring multiple aerotaxis receptors, where typically only a subset is verified to be involved in aerotaxis, leaving the roles of the remaining receptors unresolved. For example, within the multiple bipartite chemoreceptors of *C. jejuni*, only the CetA/CetB receptor has been demonstrated to influence aerotaxis regulation (Elliott & Di Rita, 2008; Elliott et al., 2009). Similarly, out of the seven predicted aerotaxis receptors in *V. cholerae*, merely two have been confirmed to mediate the aerotactic response (Boin & Hase, 2007; Shu et al., 2022). We propose that the functions of these receptors can be explored from several perspectives. Firstly, it is essential to investigate whether these receptors are involved in recognizing additional signals, particularly in light of the PAS domain’s diverse signal recognition capabilities. Secondly, it is pertinent to examine whether these receptors function under varying environmental conditions or are regulated by specific factors. For instance, the expression of AerC in *A. brasilense* is upregulated under nitrogen-fixing conditions (Xie et al., 2010). In *P. aeruginosa*, the transcription of *aer* is modulated by the anaerobic regulator ANR, which governs arginine catabolism and nitrate reduction, and Aer-mediated aerotaxis is contingent upon the presence of ANR (Hong et al., 2004b). Thirdly, it is crucial to determine whether these receptors influence other biological processes, such as biofilm formation. For example, two predicted aerotaxis receptors in *V. cholerae* may be implicated in biofilm formation (Shu et al., 2022). Fourthly, in bacteria possessing multiple chemotaxis systems, it is imperative to identify the specific system to which the aerotaxis receptors belong, as not all chemotaxis systems present in bacterial genomes contribute to or dominate aerotactic behavior. For example, *P. syringae* pv. tabaci6605 contains seven chemotaxis gene clusters, yet only the che cluster I is primarily responsible for aerotaxis (Tumewu et al., 2021; Tumewu et al., 2022). Among the four chemotaxis systems in *A. brasilense*, the dominant histidine kinase chemotaxis system that mediates aerotaxis is Che4, while Che1 enhances motility in response to oxygen gradients, thereby facilitating aerotactic behavior (Mukherjee et al., 2016). This phenomenon may be attributed to the discrete division of labor among chemotaxis pathways, wherein the aerotaxis receptor functions exclusively as part of a specific chemotaxis pathway.

Aerotaxis is a prevalent phenomenon in nature, extensively investigated in prokaryotic microorganisms, with mounting evidence also supporting its presence in eukaryotes. In the model eukaryotic microorganism *Dictyostelium discoideum*, aerotaxis is characterized by directed cell migration toward regions of higher oxygen concentrations. Recent research suggests that this behavior facilitates the developmental processes of *D. discoideum* by enabling cells to circumvent acute hypoxic conditions, notably functioning independently of mitochondria, nitric oxide, and oxidative stress (Hirose et al., 2023; Hirose et al., 2025). Furthermore, aerotaxis has been observed in the choanoflagellate *Salpingoeca rosetta*, a close unicellular relative of animals, as well as in human breast tumor cells (Kirkegaard et al., 2016; Mikaelian et al., 2022). Collectively, these findings imply that integrating insights from diverse eukaryotic lineages may elucidate the evolutionary mechanisms underlying distinct aerotaxis strategies across Earth’s biota. Beyond their evolutionary implications, research on aerotaxis possesses considerable potential for practical applications. Numerous studies have shown that the increased expression of aerotaxis receptors can enhance redox taxis (Rebbapragada et al., 1997) or improve aerotaxis in bacterial strains (Brooun et al., 1998; Boin & Hase, 2007; Huang et al., 2024). For beneficial bacteria, genetic manipulation of the aerotaxis signal transduction pathway, such as targeted upregulation of aerotaxis receptors, could enhance environmental competitiveness, thereby facilitating host colonization and symbiotic establishment. Conversely, manipulating oxygen availability or redox homeostasis in pathogenic bacteria may reduce or eliminate their capacity for infection. In the context of environmental pollutant degradation, aerotaxis receptors exhibit considerable potential. The amino acid sequence identity between Aer2 from *Pseudomonas putida* KT2701 and Aer2 from *Pseudomonas putida* F1 is as high as 99%. These two receptors exhibit a similar domain architecture to *E. coli* Aer and belong to the classic Aer-type receptors (Sarand et al., 2008; Luu et al., 2013). They are designated “Aer2” to distinguish them from other Aer-related receptors. Beyond their role as oxygen sensors, these receptors facilitate metabolism-dependent taxis towards (methyl) phenols and phenylacetic acid, both of which are classified as environmental pollutants (Sarand et al., 2008; Luu et al., 2013). This example highlights the promise of aerotaxis receptors as biosensors for the detection of environmental pollutants.

## Supplemental Information

10.7717/peerj.21573/supp-1 Supplemental Information 1 Classification and key characteristics of bacterial and archaeal aerotaxis receptors

## Additional Information and Declarations

### Data Availability

The following information was supplied regarding data availability: This is a Literature Review
