# Supplementary material for: Bacterial aerotaxis: receptor diversity, signal transduction and ecological functions
Source: PeerJ. 2026 Jul 22;14:e21573. doi: 10.7717/peerj.21573 (PMC13401364; doi:10.7717/peerj.21573)
Supplement: Supplemental Information 1 [file peerj-14-21573-s001.docx]

**Table S1：**

**Classification and Key Characteristics of Bacterial and Archaeal Aerotaxis Receptors**

| **Receptor Type** | **Receptor Name** | **Host Strain^1^** | **Class^2^** | **Cofactor^3^** | **Proposed Function^4^** | **References** |
| --- | --- | --- | --- | --- | --- | --- |
| Energy-dependent | Tlp1 | *Azospirillum brasilense* | I | Unknown | Energy sensing; PilZ domain-c-di-GMP interaction modulates cellular navigation in oxygen gradients and wheat root colonization | *Greer-Phillips et al., 2004*; *Russell et al., 2013*; *O'Neal et al., 2017* |
|  | Tsr | *Escherichia coli* | I | Unknown | Oxygen sensing via proton motive force (PMF) monitoring | *Edwards, Johnson & Taylor, 2006* |
|  | AerA | *Pseudomonas syringae* | I | Unknown | Mediates aerotaxis; promotes early-stage host plant colonization and biofilm formation | *Tumewu et al., 2022* |
|  | Aer | *Azospirillum brasilense* | II | FAD (putative) | Senses oxygen/wheat root gradients; modulates root colonization via PilZ domain | *O'Neal et al., 2019* |
|  | CetA/CetB | *Campylobacter jejuni* | II | FAD (putative) | Bipartite receptor; senses redox state; mediates human epithelial cell invasion | *Elliott & DiRita,2008*; *Elliott et al., 2009*; *Reuter & van Vliet, 2013* |
|  | Aer | *Escherichia coli* | II | FAD | Senses intracellular redox potential; core aerotaxis receptor | *Bibikov et al., 1997*; *Rebbapragada et al.,1997*; *Edwards, Johnson & Taylor, 2006* |
|  | CetA2/CetB2 | *Magnetospirillum gryphiswaldense* | II | FAD | Bipartite receptor; oxygen sensing via intracellular redox potential monitoring; mediates bacterial magneto-aerotaxis | *Herz et al., 2025* |
|  | Aer | *Pseudomonas aeruginosa* | II | FAD (putative) | Secondary aerotaxis receptor; regulates redox-dependent colonization | *Hong et al., 2004a*; *Xie et al., 2010* |
|  | Aer1-1, Aer1-2 | *Pseudomonas chlororaphis* | II | FAD (putative) | Oxygen sensing (likely via intracellular redox potential monitoring); modulates bacterial lifecycle; enhances biocontrol efficiency in soil | *Xie et al., 2010*; *Arrebola and Cazorla, 2020*; |
|  | Aer1 | *Pseudomonas putida F1* | II | Unknown | Function uncharacterized (experimental verification needed) | *Luu et al., 2013* |
|  | Aer2 | *Pseudomonas putida F1* | II | FAD (putative) | Mediates aerotaxis and chemotaxis to phenylacetic acid |  |
|  | Aer1, Aer3 | *Pseudomonas putida KT2701* | II | FAD (putative) | Function uncharacterized (experimental verification needed) | *Sarand et al., 2008* |
|  | Aer2 | *Pseudomonas putida KT2701* | II | FAD (putative) | Mediates aerotaxis and metabolism-dependent chemotaxis to phenolic compounds and other metabolites |  |
|  | Aer | *Pseudomonas putida PRS2000* | II | FAD (putative) | Mediates aerotaxis; mechanistically similar to E. coli Aer | *Nichols & Harwood, 2000* |
|  | AerB | *Pseudomonas syringae* | II | FAD (putative) | Mediates aerotaxis; contributes to early-stage host plant colonization and biofilm formation | *Xie et al., 2010*; *Tumewu et al., 2022* |
|  | Aer1, Aer2 | *Ralstonia solanacearum* | II | FAD (putative) | Mediates aerotaxis; contributes to tomato root infection (virulence-related) | *Yao & Allen, 2007*; *Xie et al., 2010* |
|  | Aer-1, Aer-3 | *Vibrio cholerae* | II | Unknown | Function uncharacterized (experimental verification needed) | *Boin & Hase,2007* |
|  | AerC | *Azospirillum brasilense* | IV | FAD | Oxygen sensing via intracellular redox potential monitoring; modulates the assembly of polar chemotactic signaling clusters | *Xie et al., 2010*; *Ganusova et al., 2023* |
|  | Aer-2 | *Vibrio cholerae* | IV | Unknown | Mediates aerotaxis; exerts a negative effect on intestinal bacterial colonization in adult mouse intestines | *Boin & Hase,2007*; *Shu et al., 2022* |
|  | Aer5 | *Vibrio cholerae* | IV | Unknown | Function uncharacterized (experimental verification needed) | *Shu et al., 2022* |
|  | Aer6, Aer7 | *Vibrio cholerae* | IV | Unknown | May contribute to bacterial biofilm formation |  |
| Direct oxygen-sensing | DcrA | *Desulfovibrio vulgaris* | II | Heme (c-type) | May sense oxygen concentration and/or intracellular redox potential | *Fu et al., 1994* |
|  | Aer2 | *Leptospira interrogans* | II | Heme (b-type) | Mediates O₂ and CO responses in the *E. coli* chemotaxis heterologous expression system | *Orillard & Watts, 2022* |
|  | HtrVIII | *Halobacterium salinarum* | III | Heme (putative) | Mediates aerophilic response in *H. salinarum* | *Brooun et al., 1998* |
|  | Atu1027 | *Agrobacterium fabrum* | IV | Heme (putative) | Mediates aerotaxis; contributes to bacterial biofilm formation and *A. tumefaciens* virulence | *Huang et al., 2024* |
|  | IcpB | *Azorhizobium caulinodans* | IV | Heme | Mediates aerotaxis; contributes to bacterial biofilm formation and the *A. caulinodans*-*S. rostrata* symbiosis | *Jiang et al., 2016* |
|  | GCSBh | *Bacillus halodurans* | IV | Heme | Direct oxygen sensing | *Hou et al., 2001b* |
|  | HemAT-Bs | *Bacillus subtilis* | IV | Heme (b-type) | Mediates aerophilic response in *B. subtilis* | *Hou et al., 2000*; *Hou et al., 2001a* |
|  | HemAT-Hs | *Halobacterium salinarum* | IV | Heme (b-type) | Essential for aerophobic response in *H. salinarum* |  |
|  | Aer-2 | *Pseudomonas aeruginosa* | IV | Heme (b-type) | Oxygen sensing; modulates *P. aeruginosa* virulence | *Hong et al., 2004a*; *Garcia et al., 2017*; *García-Fontana et al., 2019* |
|  | Aer2 | *Vibrio cholerae* | IV | Heme (b-type) | Senses oxygen via two PAS domains in the *E. coli* chemotaxis heterologous expression system | *Greer-Phillips et al., 2018* |
|  | Aer2 | *Vibrio vulnificus* | IV | Heme (b-type) | Mediates O₂ responses in the *E. coli* chemotaxis heterologous expression system | *Stuffle et al., 2022* |

Notes:

1. All strains are Gram-negative bacteria except *Bacillus subtilis* (Gram-positive) and *Halobacterium salinarum* (archaea).

1. Class I-IV refer to the structural classification of chemoreceptors (Zhulin et al., 2001): I = 1-2 transmembrane helices + periplasmic ligand-binding domain (LBD); II = 2 transmembrane helices + intracellular LBD; III = variable transmembrane helices, no periplasmic LBD; IV = entirely intracellular, no transmembrane helices.
2. "Putative" indicates the cofactor is predicted by sequence homology but not experimentally verified.
3. "Function uncharacterized (experimental verification needed)" indicates the receptor function is predicted by sequence homology but not validated by experimental assays.
